# *In Vitro* and *In Vivo* Anti-Inflammatory Activities of Benjakul: A Potential Medicinal Product from Thai Traditional Medicine

**DOI:** 10.1155/2020/9760948

**Published:** 2020-07-14

**Authors:** Pranporn Kuropakornpong, Arunporn Itharat, Sumalee Panthong, Seewaboon Sireeratawong, Buncha Ooraikul

**Affiliations:** ^1^Graduate School, Faculty of Medicine, Thammasat University, Pathum Thani 12120, Thailand; ^2^Department of Applied Thai Traditional Medicine, Faculty of Medicine, Thammasat University, Pathum Thani 12120, Thailand; ^3^Center of Excellence in Applied Thai Traditional Medicine Research (CEATMR), Thammasat University, Pathum Thani 12120, Thailand; ^4^Department of Pharmacology, Faculty of Medicine, Chiang Mai University, Chiang Mai 50200, Thailand; ^5^Department of Agricultural Food and Nutritional Science, Faculty of Agricultural Life and Environmental Sciences, University of Alberta, Edmonton, AB T6G 2P5, Canada

## Abstract

Benjakul (BJK) is a Thai traditional remedy consisting of five plants: *Piper chaba* Hunt., *Piper sarmentosum* Roxb., *Piper interruptum* Opiz., *Plumbago indica* Linn., and *Zingiber officinale* Roscoe. It is used as a first-line drug to balance patient's symptoms before other treatments. BJK ethanolic extract has been reported to show anti-inflammatory activity through various mediators, e.g., nitric oxide, TNF-*α*, IL-1*β*, and IL-6. Therefore, BJK could serve as a potential novel anti-inflammatory herbal medicine. However, studies on prostaglandin E2 (PGE2), one of the key mediators in acute inflammation, and anti-inflammation in animal models (*in vivo*) have not been done. This study investigated the anti-inflammatory activity of BJK extract and some of its chemical compounds against PGE2 production in murine macrophage (RAW 264.7) cell line and two *in vivo* models of anti-inflammatory studies. Ethanolic extract of BJK (BJK[E]) showed high inhibitory activity against PGE2 production with an IC_50_ value of 5.82 ± 0.10 *μ*g/mL but its water extract (BJK[W]) was inactive. Two chemicals from BJK[E], i.e., plumbagin and myristicin, which served as biological markers, showed strong activity with IC_50_ values of 0.08 ± 0.01 and 1.80 ± 0.06 *μ*g/mL, respectively. BJK[E] was administered both topically and orally to rats inhibited with inflammation induced by ethyl phenylpropiolate (rat ear edema model) and carrageenan (hind paw edema model). Moreover, the biological activity of BJK extract did not reduce after six-month storage under accelerated condition (40°C, 75% RH). This indicated its stability and a 24-month shelf-life under normal condition. These results supported not only the use of BJK in Thai traditional medicine but also the possibility of further development of phytopharmaceutical products from BJK.

## 1. Introduction

Inflammation of muscles can affect people of all ages. To mitigate or reduce pain, over-the-counter (OTC) or prescription drugs such as nonsteroidal anti-inflammatory drugs (NSAID) or corticosteroids pain relievers are recommended. Unfortunately, some of these drugs have some short-term or long-term negative side effects such as bleeding, indigestion, heart problems, and kidney issues. Some, such as opioids, may cause serious addiction. Therefore, more and more patients are turning to natural products to manage their pain, resulting in increased efforts to develop natural anti-inflammatory medicines.

Thailand's National List of Essential Medicines (NLEM) has approved six traditional remedies as medicines for muscle and joint pain. Each of the remedies contains a complex mixture of more than five plants, which in turns imposes limitations on the use of these traditional remedies. There are some powerful topical anti-inflammatory products which contain a single plant extract such as chili. Unfortunately, it also causes skin irritation. It is possible that, by using a combination of herbs, negative side effects of active ingredients are kept to minimum by the supplementary herbs that may appear superfluous. Therefore, the use of a combination of herbs is much preferred.

Benjakul (BJK), a Thai traditional remedy for pain, is a combination of five spicy and hot plants: *Piper chaba* Hunt. (PCH), *Piper sarmentosum* Roxb. (PSR), *Piper interruptum* Opiz. (PIO), *Plumbago indica* Linn. (PIL), and *Zingiber officinale* Roscoe (ZOR). It has been used as the first drug to reduce pain and balance patients' symptoms before administering other treatments such as inflammatory and cancer treatments [1]. According to the previous studies, the ethanolic extract of BJK has been reported to show anti-inflammatory activity through nitric oxide production inhibition but the water extract did not have this activity [2]. It was also reported to have significant inhibitory activity on the release of TNF-*α*, IL-1*β*, and IL-6 from LPS-treated CaCo-2 cells [3]. Methanolic extract of PCH at the doses of 125 and 250 mg/kg body weight showed significant anti-inflammatory activity in animal study with reduction of edema (33% and 35%, respectively) [4]. PCH, PIO, and PSR showed analgesic and anti-inflammatory activities in mice [5, 6]. ZOR exhibited high inhibitory activity on nitric oxide and prostaglandin production, both *in vitro* and *in vivo* [7, 8].

Due to its simple plant combination, together with the strong anti-inflammatory activity, BJK has been of particular interest in the development of anti-inflammatory medicines. Though its anti-inflammation has been reported, studies on its effects on prostaglandin E2 (PGE2), one of the important factors in inflammation process, and *in vivo* model, have not been done.

According to traditional usage, the patients were given BJK medicine as dried powder and decoctions were prepared by the patients themselves. BJK liquor is prepared by the traditional doctors and sometimes given to patients. Both dosage forms of BJK preparations are to be taken orally. In this study, BJK was extracted with water and ethanol mimicking the methods used by Thai traditional medicine.

**Figure 1:**
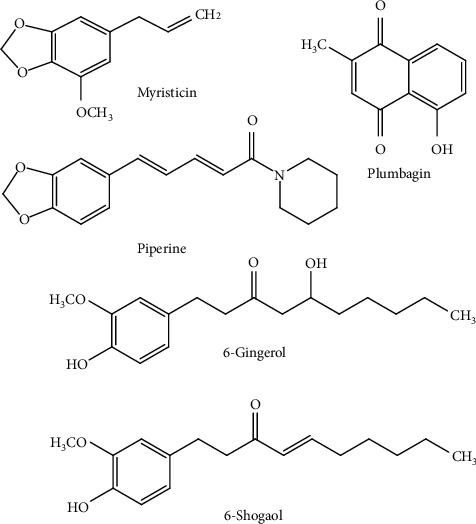
Chemical structures of pure compounds from BJK extract.

The chemical fingerprints of BJK ethanolic extract have been elucidated by high-performance liquid chromatography (HPLC). There were five markers that have been reported to be present in BJK: myristicin, plumbagin, piperine, 6-gingerol, and 6-shogaol (Figure 1). Its highest chemical marker, piperine, which is a marker for *Piper* species, was used as its analytical marker, while its biological markers for anti-inflammation were selected following the analysis of the inhibition of PGE2 production.

Chemical and biological stability are used to determine shelf-life of drugs. Heat and moisture are major factors affecting drug stability. A drug should be able to withstand storage conditions, normally ambient temperature and humidity, for a period of at least 12 months. In testing drug stability, accelerated storage test under the temperature and humidity of 40 ± 2°C and 75 ± 5% RH for at least 6 months is used to shorten the storage time before determining the change in its bioactivities. The accelerated test method provides a good estimate for drug shelf-life.

The objectives of this study were to investigate the inhibitory activity of BJK extract, its individual plant extracts, its biological markers against PGE2 production from LPS-stimulated RAW 264.7 macrophages, and its stability under the accelerated storage conditions. *In vivo* anti-inflammatory activity was also studied to confirm its effectiveness in animal model. Data obtained will be useful to the industry in the development of phytopharmaceutical products.

## 2. Materials and Methods

### 2.1. Plant Materials

Five plants ingredients of BJK were collected from several parts of Thailand and authenticated by the herbarium of Southern Centre of Thai Medicinal Plant, Faculty of Pharmaceutical Science, Prince of Songkhla University, Thailand (Table 1).

### 2.2. Chemicals

Standard compounds, myristicin, plumbagin, 6-gingerol, 6-shogaol, were purchased from Sigma-Aldrich, USA, and piperine was purchased from Merck, Thailand. HPLC-grade water, methanol, and acetonitrile were purchased from Labscan, Thailand.

Dulbecco's Modified Eagle's Medium (DMEM), fetal bovine serum (FBS), penicillin streptomycin (P/S), and 0.5% trypsin-EDTA were purchased from Gibco BRL Life Technologies (Grand Island, NY, USA). Phosphate buffer saline (PBS) was purchased from Amresco (Ohio, USA). Dimethyl sulfoxide (DMSO) was purchased from Fluka (Munich, Germany). Lipopolysaccharide (LPS) and 3-(4,5-dimethyl-2-thiazolyl)-2,5 diphenyl-2H-tetrazolium bromide (MTT) were purchased from Sigma-Aldrich Inc. (St. Louis, MO, USA). Prostaglandin E2 EIA kit monoclonal was purchased from Cayman Chemical Company (Michigan, USA).

Male Sprague-Dawley rats were obtained from National Laboratory Animal Center, Nakorn Pathom, Thailand. They were kept in a room maintained under environmental conditions of 25 ± 1°C and 12 hr dark-light cycle with free access to water and food. Rats were kept in the experimental facility for 1 week to allow them to be acclimated prior to the experiments. The Animal Ethics Committee of Faculty of Medicine, Thammasat University, Thailand, approved the experimental protocols (no. 0002/2008). After the experiments, all animals were sacrificed.

### 2.3. Preparation of Plant Extract

Each plant was cleaned, dried, and ground into coarse powder. A kilogram of BJK was prepared by mixing an equal amount of the five plants. Each plant and the BJK powder were subjected to two extraction methods. The first method was maceration with ethanol (2 : 1 weight ratio) for 3 days. The extracts were filtered and concentrated by vacuum evaporation. This process was repeated twice on the residue. The extract was designated ethanolic extract ([E]). The second method was decoction with deionized water. The aqueous extracts were dried with a lyophilizer. The extracts were designated water extract ([W]).

### 2.4. Determination of BJK Markers Using HPLC

#### 2.4.1. HPLC Systems and Conditions

BJK fingerprint was obtained by using high-performance liquid chromatography (HPLC) of the Agilent 1200 series model. The instrument is composed of a solvent degasser (G1322A), a quaternary solvent pump (G1311A), an auto sampler (G1329A), a column thermostat (G1316A), and a photodiode array detector (G1315D).

A reverse-phase column (Phenomenex): Luna® 5 *µ*m C18, 100 Å, 250 × 4.6 mm was used as the stationary phase. Samples of 10 *µ*l were injected and eluted with a gradient system composed of water and acetonitrile. The analyses of markers were performed at 210 nm (myristicin, 6-shogaol, and 6-gingerol), 254 nm (plumbagin), and 340 nm (piperine).

#### 2.4.2. Standard Preparation

Two mg of standard was weighed into a volumetric flask, using methanol as a solvent. Seven concentrations (400, 200, 150, 50, 25, 10, and 1 *µ*g/mL) of standard were prepared to produce a standard curve.

#### 2.4.3. Sample Preparation

Ten mg of BJK extract was dissolved in 1 mL of methanol to make 10 mg/mL concentration and filtered through a 0.45 *µ*m membrane filter.

### 2.5. *In Vitro* Anti-Inflammatory Activity of BJK Extract and Its Plant Components by PGE2 Inhibition Assay [9]

BJK and each plant ingredient were subjected to two extraction methods which were maceration with 95% ethanol as ethanolic extract [E] and decoction as water extract [W] (as described in Section 2.3) and tested for the *in vitro* anti-inflammatory activity by PGE2 inhibition assay as follows:

  Step 1: the RAW 264.7 cells in Dulbecco's Modified Eagle's Medium (DMEM) were seeded into 96-well plates with density of 1 × 10^5^ cells/well and incubated at 37°C, 5% CO_2_, for 24 hr. Subsequently, the old medium was replaced with fresh medium containing 2 *μ*g/mL of LPS; then test samples were added and incubated at 37°C, 5% CO_2_, for 24 hr.
   Step 2: PGE2 production by enzyme-linked immunosorbent assay (ELISA) was determined by transferring 50 *µ*L of supernatant into a goat anti-mouse IgG coated plate. After that, 50 *μ*L each of prostaglandin E2 AChE tracer and prostaglandin E2 monoclonal antibody was added to each well and incubated at 4°C for 18 hr. The plate was washed with wash buffer; then Ellman's reagent was added and incubated at 37°C for 60–90 minutes. The optical density (OD) was measured at 412 nm. The 50% inhibitory concentration (IC_50_) was calculated. All data were expressed as mean ± standard error of means (SEM).
  Step 3: RAW 264.7 cells viability was determined by the MTT assay. After separating supernatant from the incubated plate (in Step 1), 5 mg/mL MTT solution was added to each well and incubated at 37°C, 5% CO_2_, for 2 hr. Subsequently, the old medium was removed and 100 *μ*L of 0.04 molar of hydrochloric acid (HCl) in isopropanol was added to dissolve the formazan product. The OD was measured at 570 nm. Percentage of cell survival should be above 70% compared to control.

### 2.6. *In Vivo* Anti-Inflammatory Activity Testing

BJK[E] which showed high *in vitro* anti-inflammatory activity was then tested for the *in vivo* anti-inflammatory activities.

#### 2.6.1. Ethyl Phenylpropiolate- (EPP-) Induced Ear Edema in Rats [10]

Male rats (40–60 g) were divided into five groups of three rats each (*n* = 6). Twenty *µ*L of acetone, phenylbutazone (final concentration of 1 mg/ear), and three doses of BJK ethanolic extracts (BJK[E]) at various final concentrations of 1, 2, and 4 mg/ear were applied topically to the control, reference, and test group, respectively. Ethyl phenylpropiolate (EPP) was then applied topically (1 mg/20 *µ*L/ear) on the inner and outer surface of both ears to induce edema. The thickness of each ear was measured with a digital Vernier caliper at 15, 30, 60, and 120 min after edema induction.

Results were expressed as mean ± SEM compared with the control group at each time interval. Statistical significance was determined by one-way analysis of variance (ANOVA) at the 95% confidence interval.

#### 2.6.2. Carrageenan-Induced Paw Edema in Rats [11]

Male rats (100–120 g) were divided into five groups of six each. Each group was orally administered 5% Tween 80, aspirin (300 mg/kg), and various doses of BJK ethanolic extract (BJK[E]) (300, 600, and 1,200 mg/kg) as the control, reference, and test group, respectively. Samples were feed one hour prior to carrageenan injection. A 0.05 mL of 1% carrageenan in sterile normal saline solution (NSS) was injected intradermally into the plantar side of the right hind paw of the rats. The edema volumes were determined using a plethysmometer (model 7140, Ugo Basile, Italy) at 1, 3, and 5 hr after carrageenan injection.

Results were expressed as mean ± SEM compared with the control group at each time interval. Statistical significance was determined by one-way analysis of variance (ANOVA) at the 95% confidence interval.

### 2.7. Stability Testing of BJK Extract under the Accelerated Conditions

BJK extracts were kept at 40 ± 2°C and 75 ± 5% RH for 6 months [12]. An aliquot of 1 gram was taken every 30 days and tested for inhibition activity against PGE2 production. One-way ANOVA was used to determine the difference between day 0 and other days.

## 3. Results and Discussion

### 3.1. Determination of BJK Markers Using HPLC

**Figure 2:**
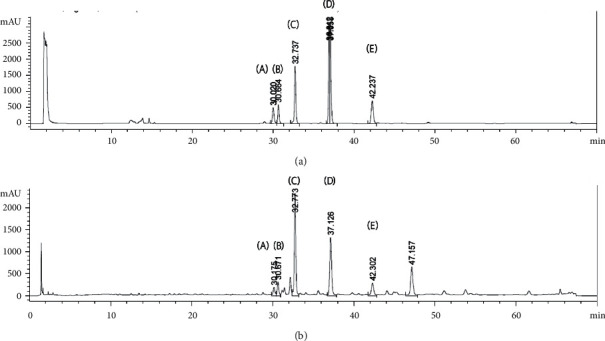
HPLC profile of five standards (a) and BJK extract (b) detected with diode array at 210 nm. Plumbagin (A), 6-gingerol (B), Piperine (C), myristicin (D), and 6-shogaol (E).

There were 5 reported markers in BJK extract, i.e., myristicin, plumbagin, piperine, 6-gingerol, and 6-shogaol (Figure 1). HPLC chromatogram at 210 nm showed the presence of these markers in BJK extract (Figure 2). The peak identity was confirmed by the retention time and the scanned spectrum of each peak matching to that of pure standard. The quantitative analysis of each marker was done at different wavelengths that provided the best selectivity, i.e., 6-gingerol, myristicin, and 6-shogaol at 210 nm; plumbagin, at 254 nm; and piperine at 340 nm.

### 3.2. *In Vitro* Anti-Inflammatory Activity of BJK Extract and Its Plant Components by PGE2 Inhibition Assay

**Figure 3:**
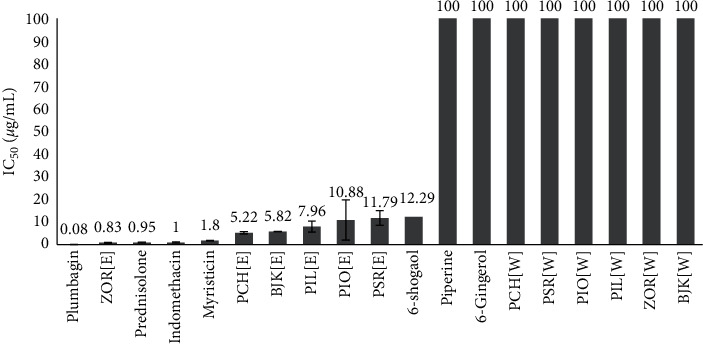
IC_50_ of BJK extract, its components, and pure compounds on the inhibition of PGE2 production. BJK: Benjakul, PCH: *Piper chaba* Hunt., PSR: *Piper sarmentosum* Roxb., PIO: *Piper interruptum* Opiz., PIL: *Plumbago indica* Linn., and ZOR: *Zingiber officinale* Roscoe. The letter in refers to the extraction method; [E]: ethanolic extract and [W]: water extract.

The results showed that all water extracts tested, including piperine and 6-gingerol, did not have the PGE2 production inhibition activity. Plumbagin was the most active compound with IC_50_ value of 0.08 ± 0.01 *µ*g/mL which was better than the standard drugs prednisolone and indomethacin (IC_50_ values of 0.95 and 1.00 *µ*g/mL, respectively). Myristicin was the second active compound in BJK with IC_50_ value of 1.80 ± 0.06 *µ*g/mL while 6-shogaol was the third active compound with IC_50_ value of 12.29 ± 0.02 *µ*g/mL. Comparison between the activities of various ethanolic extracts revealed that ZOR was the most active extract with IC_50_ value only 0.83 ± 0.26 *µ*g/mL which was better than prednisolone and indomethacin. PCH and BJK were the second and third active extracts with IC_50_ values of 5.22 ± 0.53 and 5.82 ± 0.10 *µ*g/mL, respectively. The ethanolic extracts of PIL, PIO, and PSR were less active with IC_50_ values of 7.96 ± 2.44, 10.88 ± 8.88, and 11.79 ± 3.20 *µ*g/mL, respectively (Table 2, Figure 3). The Thai traditional medicine [TTM] is also known to have used ginger for topical treatment of joint inflammation, and, according to the theory of TTM, the herbs that generate heat are useful for Vata (a wind element) problem such as arthritis. Other plant components also contained some inhibition activity on PGE2 production. Increment of ZOR and PCH composition in BJK could possibly promote the anti-inflammatory activity of BJK.

These results support the use of BJK as anti-inflammation drug in Thai traditional medicine. BJK contains more than one bioactive compound that exerted anti-inflammatory activity, which may explain the decrease in its toxicity against normal cells as compared to the extracts from individual plants. A previous study showed that plumbagin decreased the expression of NF-*κ*B-regulated gene products that play a role in cell proliferation, antiapoptosis, and inflammation [13], including decreasing the expression of COX2 in mice with prostate cancer [14]. Myristicin also showed high inhibitory activity on PGE2 release. A previous report showed that myristicin significantly decreased nitric oxide, inflammatory cytokines production, and growth factor in macrophage [15]. Even though the mechanism of BJK and its compounds on PGE2 inhibition in this study was not clear, from previous reports, BJK showed significant inhibitory activity on IL-6, IL-1*β*, and nitric oxide. [2, 3]. Future work should focus on the selective COX-2 inhibition, which is the key enzyme of PGE2 and IL-6 production and other inflammatory mediators in *in vivo* model.

### 3.3. *In Vivo* Anti-Inflammatory Activity of BJK Extract

#### 3.3.1. Ethyl Phenylpropiolate- (EPP-) Induced Ear Edema in Rats

**Figure 4:**
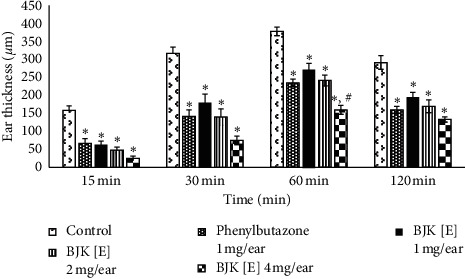
The thickness of ear at 15, 30, 60, and 120 min after edema induction. ^∗^Significant difference from the control group (*P* < 0.05). ^#^Significant difference from phenylbutazone (*P* < 0.05) according to one-way ANOVA with Tukey's HSD test. BJK [E]: Benjakul ethanolic extract.

The EPP-induced rat ear edema is conventionally used to estimate anti-inflammatory activity of test substances during the acute phase of inflammation. The inflammatory mediators released in EPP-induced ear edema model are histamine, serotonin, bradykinin, and prostaglandins (PGs); these mediators are capable of promoting vasodilatation and increasing vascular permeability as well as synergistically producing edema [10,16]. *In vivo* study on anti-inflammatory activity using EPP-induced rat ear edema showed that BJK ethanolic extract (BJK[E]) at 1, 2, and 4 mg/ear could reduce edema with significant difference from control. At the dose of 4 mg/ear, BJK[E] could reduce edema better than phenylbutazone with significant difference at 60 min (Figure 4).

Topical administration of BJK[E] showed anti-inflammatory activity by inhibiting the inflammatory mediators of the acute phase of inflammation via inhibition of release and/or formation of inflammatory mediators involved in edema formation which was related to the *in vitro* results.

#### 3.3.2. Carrageenan-Induced Paw Edema in Rats

**Figure 5:**
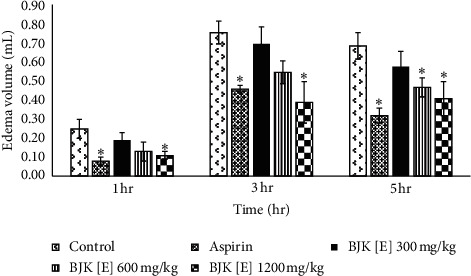
The edema volume of rat paw at 1, 3, and 5 hr. ^*∗*^Significant difference from the control group (*P* < 0.05) according to one-way ANOVA with Tukey's HSD test. BJK [E]: Benjakul ethanolic extract.

The *in vivo* anti-inflammatory activity of BJK extract was confirmed by the carrageenan-induced paw edema model. Several inflammatory mediators, for example, histamine, serotonin, kinins, PGs, complement, and proinflammatory cytokines, play a major role in paw edema caused by carrageenan [17, 18]. The initial phase is caused by the release of histamine and serotonin followed by the release of bradykinin during 1–2 hr after carrageenan injection [19]. The release of PGs is closely associated with leukocytes migration to the inflamed site. The presence of PGs, particularly PGE_2_, in the inflammatory exudates from the injected foot can be demonstrated at 3 hr and thereafter [20]. Oral administration of BJK[E] at the dose of 1,200 mg/kg could reduce the swollen of carrageenan-induced paw edema with significant difference from control at 1, 3, and 5 hr and was not different from the standard drug, aspirin. However, the BJK extract at 300 mg/kg showed no activity at any time interval (Figure 5).

Oral administration of BJK[E] at the concentration of 1,200 mg/kg showed the anti-inflammatory activity by inhibiting the acute phase of inflammation. Based on the inhibitory effect of the BJK[E] seen at the 3^rd^ hr and 5^th^ hr, it suggests that the main mechanism of action may be due to inhibition of PGs synthesis. Moreover, the inhibitory effect of the BJK[E] may partly involve other acute inflammatory mediators such as histamine, serotonin, bradykinin, and proinflammatory cytokines which are released during the 1^st^ hr after carrageenan injection.

### 3.4. Stability Testing of BJK Extract under the Accelerated Conditions

No physical difference between the normal and stored BJK extract samples was detected after the completion of the accelerated stability test. Anti-inflammatory activity of the samples was unchanged up to day 120 and then increased significantly on day 180 with IC_50_ values decreasing from about 4.3 to 1.76 ± 0.15 *µ*g/mL. Despite the increasing activity, the reductions in the content of the three markers were observed: piperine and myristicin content remained 84.88% and 91.53%, respectively, on day 180 while plumbagin, the strongest active marker, has not been detected since day 60 (Table 3). The loss of plumbagin could be due to its sublimation property even at room temperature. The results suggested that the biological activity of BJK extract was related to other substances more than those selected markers in this study. It also implied that for stability study of BJK extract, the biological activity should be performed instead of the chemical analysis. Our study has demonstrated that BJK extract was stable at accelerated conditions and could have 24-month shelf-life at room temperature.

## 4. Conclusion

Our findings strongly confirmed the anti-inflammatory activity of BJK and its plant components especially ZOR and PCH. Increasing the composition of these two plants may increase the anti-inflammatory activity of BJK. Plumbagin and myristicin were the active compounds found in BJK and exerted stronger activity than standard drugs. The results supported its traditional use as an anti-inflammatory drug. BJK showed potent activity in both *in vitro* and *in vivo* studies, which persisted through six-month accelerated conditions. Our results also suggested that biological activity, rather than chemical analysis, should be performed in BJK stability testing. It can also be concluded that BJK extract could have a 24-month shelf-life under normal room condition. These results supported not only the use of BJK in Thai traditional medicine but also the possibility of further development of phytopharmaceutical products from BJK. This is the first report *on in vivo* anti-inflammatory activity of BJK[E] by topical administration.

## Figures and Tables

**Table 1:** Plant materials used in BJK remedy.

	General name	Code	Part of use in BJK	Voucher specimen
1	*Piper chaba* Hunt.	PCH	Fruit	SKP 146160301
2	*Piper sarmentosum* Roxb.	PSR	Root	SKP 146161901
3	*Piper interruptum* Opiz.	PIO	Stem	SKP 146160901
4	*Plumbago indica* Linn.	PIL	Root	SKP 148160901
5	*Zingiber officinale* Rosc.	ZOR	Rhizome	SKP 206261501

**Table 2:** Percentage of inhibition at various concentrations and IC_50_ values of the ethanolic and water extract of plant component in BJK preparation on PGE2 production.

Plant	Extract	% inhibition at various concentrations (mean + SEM) (% survival of cells at the highest concentration)								IC_50_(*µ*g/mL)
		100 *µ*g/mL	50 *µ*g/mL	30 *µ*g/mL	20 *µ*g/mL	15 *µ*g/mL	10 *µ*g/mL	1 *µ*g/mL	0.1 *µ*g/mL	
PCH.	Ethanol			86.54 ± 7.30 (77.33 ± 3.64)			73.72 ± 0.03	36.38 ± 1.77	35.91 ± 0.82	5.22 ± 0.53
	Water	10.92 ± 4.18 (97.56 ± 2.06)								>100

PSR.	Ethanol	87.50 ± 4.21 (98.04 ± 4.08)	95.36 ± 1.33				47.46 ± 5.52	30.09 ± 2.37		11.79 ± 3.20
	Water	5.09 ± 2.90 (59.23 ± 4.24)								>100

PIO	Ethanol			81.58 ± 2.67 (79.14 ± 2.62)			41.39 ± 2.53	15.60 ± 5.41	−6.92 ± 5.97	10.88 ± 8.88
	Water			10.28 ± 2.69 (74.14 ± 2.44)						>100

PIL.	Ethanol				98.70 ± 1.78 (83.35 ± 6.11)		66.64 ± 17.96	1.02 ± 14.62	−9.47 ± 20.61	7.96 ± 2.44
	Water	31.15 ± 3.84 (93.41 ± 2.13)								>100

ZOR	Ethanol					75.21 ± 15.07 (81.06 ± 6.28)	79.86 ± 13.27	57.99 ± 11.21	18.40 ± 9.33	0.83 ± 0.26
	Water							11.72 ± 1.78 (80.69 ± 3.10)		>100

BJK	Ethanol				83.20 ± 2.76 (95.41 ± 1.2)		80.16 ± 2.25	13.45 ± 1.56	8.77 ± 2.02	5.82 ± 0.10
	Water						9.67 ± 0.79 (75.40 ± 4.1)	6.66 ± 3.29	8.21 ± 2.98	>10

BJK: Benjakul, PCH: *Piper chaba* Hunt., PSR: *Piper sarmentosum* Roxb., PIO: *Piper interruptum* Opiz., PIL: *Plumbago indica* Linn., and ZOR: *Zingiber officinale* Roscoe.

**Table 3:** Marker contents and anti-inflammatory activity of BJK extract after the stability test.

Testing	Marker content mean (mg/g) ± SD						IC_50_ on PGE2 inhibition^d^(mean *µ*g/mL ± SEM)
	Plumbagin		Piperine^b^		Myristicin^c^		
	Content^a^(mg/g)	% remaining	Content^b^(mg/g)	% remaining	Content^c^(mg/g)	% remaining	
BJK day 0	0.86 ± 0.03	100	104.83 ± 10.11	100	4.03 ± 0.09	100	4.42 ± 0.34
BJK day 15	0.83 ± 0.01^*∗*^	97.35	94.20 ± 2.48	89.86	2.99 ± 0.05^*∗*^	74.25	5.24 ± 0.23
BJK day 30	0.81 ± 0.01^*∗*^	94.81	105.88 ± 0.40	101.00	3.54 ± 0.11^*∗*^	87.92	4.64 ± 0.18
BJK day 60	ND	ND	84.52 ± 1.36^*∗*^	80.62	3.10 ± 0.04^*∗*^	76.85	4.23 ± 1.10
BJK day 90	ND	ND	93.12 ± 0.76^*∗*^	88.83	3.02 ± 0.02^*∗*^	74.91	4.18 ± 0.19
BJK day 120	ND	ND	95.18 ± 0.65	90.79	3.28 ± 0.06^*∗*^	81.33	4.47 ± 0.72
BJK day 180	ND	ND	88.98 ± 1.65^*∗*^	84.88	3.69 ± 0.03^*∗*^	91.53	1.76 ± 0.15^*∗*^

^*∗*^ *P* < 0.05; each sample was compared with BJK day 0 using One-way ANOVA statistical analysis. ^a^Data were calculated following the standard linear equation: *y* = 52.702x–333.81, *R*^2^ = 0.9982. ^b^Data were calculated following the standard linear equation: *y* = 22.452x–1500.8, *R*^2^ = 0.9997. ^c^Data were calculated following the standard linear equation: *y* = 7.0348x–117.11, *R*^2^ = 0.9991.

## Data Availability

The data used to support the findings of this study are included within the article.
